# Perceptions of good death and attitudes toward dignified death and euthanasia among nursing students in Türkiye: a cross-sectional descriptive-correlational study

**DOI:** 10.1186/s12909-025-08435-6

**Published:** 2025-12-17

**Authors:** İpek Köse Tosunöz, Gamze Çi̇nçi̇noğlu

**Affiliations:** 1 Faculty of Health Sciences, Department of Nursing, Hatay Mustafa Kemal University, Hatay, Türkiye; 2 Faculty of Health Sciences, Nursing Department, Hatay Mustafa Kemal University, Hatay, Türkiye

**Keywords:** Death, Dignified death, Euthanasia, Good death, Nursing students

## Abstract

**Background:**

Nursing students’ perceptions and attitudes towards death and end-of-life care are of critical importance for the quality of end-of-life care and the professional roles they will undertake in this regard in the future. This study aimed to examine nursing students’ perceptions of good death, their attitudes towards the principles of dignified death, euthanasia, and the relationship between them.

**Methods:**

The sample of this cross-sectional descriptive-correlational study consisted of 259 nursing students in the 2nd, 3rd, and 4th year at two state universities in Türkiye during the 2023–2024 academic year. Data were collected with the “Personal Information Form,” “Good Death Scale,” “Assessment Scale of Attitudes toward the Principles of Dying with Dignity (ASAPDD)” and “Euthanasia Perception Scale.”

**Results:**

Nursing students’ ASAPDD (49.21 ± 6.42) and Good Death Scale (57.18 ± 2.65) mean scores were above average. Regarding euthanasia, the positive (52.89 ± 16.87) and cultural (11.50 ± 2.90) sub-dimensions were above average, the negative sub-dimension was moderate (28.44 ± 8.47), and the penal (11.64 ± 5.17) and opportunistic (7.05 ± 2.84) sub-dimensions were below average.

**Conclusions:**

Nursing students generally approached dignified and good death positively and held supportive views on euthanasia, particularly in its positive and cultural dimensions. Nursing students did not support punitive approaches toward euthanasia and the idea of deriving personal profit from its implementation. As students’ attitudes toward the principles of dignified death increased, their perceptions of a good death and positive and cultural attitudes toward euthanasia also increased. As students’ perceptions of a good death increased, cultural attitudes also increased. These results highlight the importance of incorporating ethics and end-of-life care education into the nursing curriculum.

**Clinical trial number:**

Not applicable.

**Supplementary Information:**

The online version contains supplementary material available at 10.1186/s12909-025-08435-6.

## Introduction

Death is a universal truth shared by all living organisms, feared and avoided to be talked about, and is the inevitable end of life [1, 2]. Technological and scientific developments have changed the way death occurs today, causing the life-and-death process to be prolonged [3]. The aging society and increasing chronic disease burden have increased interest in the concept of good death [4]. A good death is a process where an individual’s autonomy is respected, they can make decisions about treatment options, manage their emotions, and have pain and other symptoms effectively controlled [5, 6]. The concept of dying with dignity is discussed in various fields such as psychology, sociology, medicine, and nursing, and different definitions are made. The attributes of dying with dignity in end-of-life nursing care include human dignity and holistic care [7]. The World Health Organization accepted that patients who cannot be treated have the right to die with dignity [2, 8]. Death with dignity aims to protect the patient from unnecessary stress, pain, suffering, and traumatic treatment interventions, to feel valued, to be with their loved ones in the last stages of their life, and to have the opportunity to say goodbye to them [3, 8]. Protecting the dignity of dying patients increases nurses’ self-esteem, contributes to their personal and professional development, helps develop a positive attitude towards end-of-life care, and increases the value of care for patients [7].

Euthanasia, which comes from a Greek word meaning a good death, is commonly called mercy killing [9, 10]. Euthanasia is the intentional act of ending a person’s life, or assisting them in doing so, at their explicit request, to relieve unbearable and incurable suffering [9, 11, 12]. Patients who experience loss of dignity during the death process may request euthanasia or be inclined to request it [9]. Euthanasia is a frequently discussed topic in medical, ethical, religious, legal, and sociological contexts at the universal level [9, 11]. Today, when modern medicine prolongs the dying process, the necessity of rethinking the ethical dilemma between the sanctity of life and the autonomy of terminally ill patients is emphasized [12]. Supporters of euthanasia focus on the principle of respect for autonomy and argue that an early, dignified death within the framework of the principle of beneficence is superior to an unqualified life [13]. In addition to euthanasia, related concepts such as “assisted dying” and “assisted suicide” are also discussed. Assisted dying, a term used particularly in New Zealand, refers to practices aimed at ending the life of a suffering person. Assisted dying includes the provision of medication to a person for self-administration as well as the administration of medication by a health practitioner, in both cases causing death. “Assisted suicide,” on the other hand, is used to describe the distribution of a drug that can cause the death of a person and is used in Switzerland, Austria, and the Netherlands. Another related concept is “Medical Assistance in Dying (MAID),” which is used in Canada. MAID is a process that allows someone who is found eligible to receive assistance from a medical practitioner in ending their life [14]. While euthanasia is prohibited in most countries, it is legally permitted under certain conditions in some. Only a limited number of countries worldwide have legalized both euthanasia and physician-assisted suicide. The Netherlands (2002) and Belgium (2002) were the first to legalize both practices, followed by Luxembourg (2009), Canada (2016), Spain (2021), New Zealand (2021), all Australian states (2018–2023), and Portugal (2023). In contrast, euthanasia alone is legally permitted in Colombia (2015), whereas only physician-assisted suicide is allowed in Austria, Switzerland, Germany, and several states in the United States. In Turkey, however, euthanasia remains strictly prohibited [9, 11, 12, 14].

The dying individual and their family have various physical, psychological, social, and spiritual needs [1]. Nurses play an important role in meeting the needs of dying patients and ensuring that they have a peaceful, dignified, and good death experience [3, 6]. The roles of nurses in assisted dying vary across countries. In Canada, nurse practitioners are legally authorized to take an executive role within the framework of MAiD. According to legal regulations, they may either directly administer the life-ending medication to eligible patients or provide it for self-administration. In contrast, in most other countries, nurses do not take an executive role; rather, they play important supportive roles in the process, such as providing palliative and end-of-life care and supporting patients and their families [14]. The main purpose of end-of-life care is to comfort the patient physically, psychologically, socially, and spiritually and to ensure they experience a good death process [5, 15]. The care nurses provide to dying patients is influenced by their attitudes toward death [5]. Increasing the perception of a good death increases positive attitudes toward the care of dying patients and clinical competence in end-of-life care [16, 17]. To ensure a good and dignified dying process for patients, nurses should adopt the principles of a dignified death, provide care following these principles, and be aware of the concept and criteria of a good death [2, 3, 5, 8]. Today’s student nurses will be tomorrow’s nurses who take on the role of end-of-life care [4]. Nursing students who encounter terminal patients during their clinical practice and will be responsible for end-of-life care in their professional lives must be prepared to play a supportive role in caring for dying patients. Therefore, it is important to understand nursing students’ perceptions and attitudes toward death and death-related processes [1, 9]. To improve the quality of end-of-life care, nursing educators need to understand how students perceive good death, dignified death, and euthanasia [4]. Attitudes and perceptions towards death-related concepts may vary from culture to culture [6]. Limited studies are addressing the issues of good death, principles of dignified death, and euthanasia among nursing students in Türkiye. Studies on euthanasia in the literature are mostly conducted using questionnaire forms created in line with the literature rather than valid and reliable scales [18, 19]. In contrast, this study aimed to examine euthanasia in relation to the concepts of good death and dignified death using a validated scale, thus providing more objective and comparable data to the field. This study aims to investigate the nursing students’ perceptions of good death, their attitudes towards the principles of dignified death and euthanasia, and the relationship between them. Results will contribute to nursing education planning by understanding nursing students’ perceptions and attitudes toward good death, dignified death, and euthanasia. This will also improve the quality of end-of-life care in the long term.

### Research questions

1. What are the perceptions of nursing students regarding the concept of a good death?
2. What are the attitudes of nursing students toward the principles of dying with dignity?
3. What are the attitudes of nursing students toward euthanasia?
4. Do nursing students’ perceptions of good death and their attitudes toward the principles of dignified death and euthanasia differ according to their sociodemographic and end-of-life care–related characteristics?
5. What is the relationship between nursing students’ perceptions of good death and their attitudes toward the principles of dying with dignity and euthanasia?

## Methods

### Settings

The study was conducted at two public universities in Türkiye’s southern and northwestern provinces.

## Design

A cross-sectional descriptive-correlational study design was used.

## Population and sample

The study population consisted of 2nd, 3rd, and 4th-year students at two public universities in two cities of Turkiye in the 2023–2024 academic year (*n* = 603). Inclusion criteria were: (a) being a 2nd, 3rd, or 4th-year nursing student, (b) having completed at least one clinical practice, and (c) voluntarily agreeing to participate. Exclusion criteria were being a 1st-year student and not volunteering to participate. Considering that clinical practice experience shapes students’ perceptions of death not only at a theoretical level but also through direct clinical encounters, first-year students, who had not yet gained sufficient clinical practice experience, were not included in the study. The study sample size was calculated using the known universe sampling formula. To ensure a 95% confidence level with a 5% margin of error, at least 235 students needed to be included in the study [20]. The study population was stratified according to university (south and northwest regions) and study year (2nd, 3rd, and 4th years). After stratification, participants were selected through convenience sampling within each stratum. It was planned to sample at least 122 students (32 2nd-year, 45 3rd-year, and 45 4th-year students) from the university in the south and 113 students (39 2nd-year, 39 3rd-year, and 35 4th-year students) from the university in the northwest of Turkey. The study sample consisted of 259 nursing students (140 from the south and 119 from the northwestern region).

## Data collection tools

In the study, a questionnaire consisting of a personal information form and three scales was used as the data collection tool. Data were collected using the “Personal Information Form” developed by the researchers based on the literature [1, 4, 6, 9]; the “Good Death Scale,” for which the Turkish validity and reliability study was conducted by Fadıloğlu and Aksu Menekli (2013) [5]; the “Assessment Scale of Attitudes toward the Principles of Dying with Dignity (ASAPDD),” for which the Turkish validity and reliability study was conducted by Duyan (2014) [2]; and the “Euthanasia Perception Scale” developed by Filiz et al. [11].

*Personal Information Form*, created by researchers using literature [1, 4, 6, 9], consists of seven questions about students’ sociodemographic characteristics (age, study year, and gender) and characteristics associated with end-of-life care (knowledge about end-of-life care, caring for a dying patient, encountering death in clinical practice, and emotions during the first encounter with death). The age variable was continuous, while the other variables were categorical. Students were asked about their emotions when they first encountered death and were informed that they could provide multiple responses (See Supplementary material-1).

*The Good Death Scale* was developed by Schwartz et al. [21] to determine nurses’ views on the concept of good death [21]. Fadıloğlu and Aksu Menekli conducted the Turkish validity and reliability study of the scale [5]. The scale is a 4-point Likert-type scale and consists of 17 questions and 3 sub-dimensions: psychosocial and spiritual (9 questions), personal control (3 questions), and clinical control sub-dimension (5 questions). All items are scored from not at all (1) to very much (4). There are no reverse-coded expressions. The score range is 17–68. As the score increases, the perception is also higher. The total Cronbach’s Alpha coefficient of the scale was found to be 0.92 [5].

*The Assessment Scale of Attitudes toward the Principles of Dying with Dignity (ASAPDD)* was developed by Duyan [2] to assess individuals’ attitudes towards the principles of respectful death. The scale consists of a single factor, 12 items, and a five-point Likert-type scale (1: Strongly Disagree- 5: Strongly Agree). The score range is 12–60. The higher the score, the higher the level of adoption of the principles of dignified death. The Cronbach’s alpha coefficient of the scale was identified as 0.89 [2].

*The Euthanasia Perception Scale* was developed by Filiz et al. [11] to examine the attitudes of Turkish society towards euthanasia. The scale is a 5-point Likert type (1: strongly disagree- 5: strongly agree) consisting of 38 items and 5 sub-dimensions namely “positive attitude” (1–18. items), “negative attitude” (19–27. items), “penal attitude” (28–32. items), “opportunistic attitude” (33–35. items) and “cultural attitude” (36–38. items). The Positive Attitude sub-dimension consists of items stating that euthanasia can be applied and that it is a right at least as much as the right to life. The negative attitude sub-dimension consists of items stating that euthanasia is never acceptable and that this situation is a violation of life. The punitive attitude sub-dimension includes items advocating that punishment should be imposed in case of euthanasia. The opportunistic attitude sub-dimension includes items that state that euthanasia should be implemented and that a certain amount of profit should be gained from it. The cultural attitude sub-dimension includes items that argue that culture is the effective factor in whether euthanasia is implemented or not. There are no reverse-coded items. A higher score in each sub-dimension indicates a stronger attitude represented by that sub-dimension. The total Cronbach’s Alpha coefficient of the scale was found to be 0.97 [11].

## Data collection

After obtaining the ethics committee approval and institutional permissions, data were collected via a face-to-face survey method in the spring semester of the 2023–2024 academic year, between March and May 2024, by the researchers. Due to the large number of students enrolled in the courses, data were collected by the researchers at the beginning of the basic vocational courses, in the classroom setting, with the permission of the instructors responsible for the course. To avoid any pressure on participation, course instructors were not included in the data collection process. Students were clearly informed that participation was entirely voluntary and that their failure to participate would not affect their course grades or academic standing in any way. The research process was anonymized; no names, student numbers, or any personally identifiable information were requested on the questionnaires. The researchers were present in the classroom only to distribute the questionnaires and returned at the end of the allotted time to collect them. Students completed the questionnaires individually at their own desks in the classroom setting. To minimize potential biases that might arise from the researchers being known to some students, various precautions were taken, such as not involving the instructors of the courses in which the data were collected, making participation voluntary, ensuring anonymity, and having students fill out the surveys individually. Completing the questionnaires took an average of 20 min.

### Data analysis

Data were analyzed through the Statistical Package for Social Sciences (22.0) program. Before the analysis, the data set was examined for missing data, erroneous data, and extreme values [22, 23]. Missing data were analyzed using the Missing Value Analysis in SPSS. The distribution of missing data in the scales was examined with the Missing Completely at Random test. The rate of missing data was less than 5%, and its distribution was random. Since the rate of missing data was low, the series mean was used to impute missing values. Subsequently, univariate and multivariate outlier analyses were conducted. The extreme values were checked considering the Z-scores of the scale scores. The data other than the ± 3 were considered extreme values [23]. Mahalanobis distance was used to detect multivariate extreme values. Seven (*n* = 7) outliers were detected and removed from the dataset. All statistical analyses were performed with a data set of 259 participants. The skewness and kurtosis values were checked for the assumption of normality, and the values were between − 1 and + 1 [24]. Number, percentage, mean, standard deviation, minimum, and maximum values were used for descriptive statistics. To analyze the differences between groups, the Independent Samples t-test and one-way ANOVA were used. The Scheffe test was performed as a post hoc test. Pearson’s correlation coefficient (r) was used to analyze the correlation of study variables. In the correlation analysis, coefficient values were interpreted as indicating a weak level of correlation between 0.00 and 0.29, a moderate level between 0.30 and 0.69, and a high level between 0.70 and 1.00 [25]. Cronbach’s alpha coefficients were calculated. *p* < 0.05 value was accepted for statistical significance.

## Ethical considerations

The ethical standards stated in the Declaration of Helsinki were complied with throughout the study [26]. Ethical approval for this study was obtained from the Hatay Mustafa Kemal University Non-Interventional Clinical Research Ethics Committee (Date: 05.02.2024 and Number: 01/28). Institutional permissions were obtained from both faculties. Permission to use the scales was obtained via e-mail from the authors who conducted the Turkish validity and reliability of the scales. Written informed consent was obtained from the students who agreed to participate in this study. The researchers informed the participants about the purpose of the study. Students were assured that their data would remain anonymous, confidential, and stored securely. At the beginning of the study, students were informed that participation was entirely voluntary, that they were free to decline participation without any negative consequences, and that their decision would not affect their courses.

## Results

### Participants’ characteristics

The study sample consisted of 259 students, 140 (54.1%) from a university in the south and 119 (45.9%) from a university in the northwest of Türkiye. Of the students, 32.8% were 2nd-year students, 33.2% were 3rd-year students, and 34% were 4th-year students. The mean age of the students was 21.52 ± 1.51, and 78.8% were female. 65.3% of the students reported that they knew end-of-life care, 68% had provided care to dying patients, and 50.2% had encountered dying patients during their clinical practice (Table 1).

**Table 1:** Descriptive characteristics of students (*n* = 259)

Variables	*n*	%
Age (years) (X̄ ± SD = 21.52 ± 1.51; Min-Max = 19–29)		
19–23	238	91.9
24–29	21	8.1
Study year		
2nd-year	85	32.8
3rd-year	86	33.2
4th-year	88	34.0
Gender		
Female	204	78.8
Male	55	21.2
Knowledge about end-of-life care		
Yes	169	65.3
No	90	34.7
Caring for a dying patient		
Yes	83	32.0
No	176	68.0
Encountering death in clinical practice		
Yes	130	50.2
No	129	49.8

**Fig. 1:**
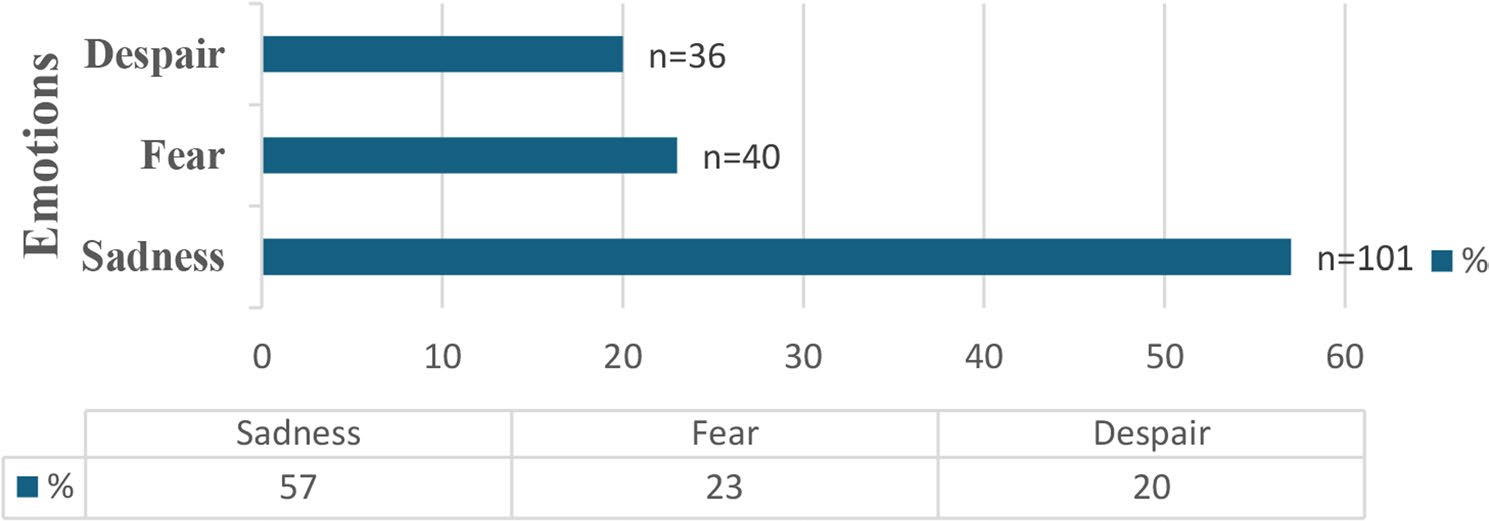
Emotions during the first encounter with death

Students stated that when they first encountered death during their clinical practice, they mostly experienced sadness (57%), fear (23%), and helplessness (20%), respectively (Fig. 1).

### Mean scores of the scales

The mean score of nursing students on the Good Death Scale was 57.18 ± 2.65 (min-max: 37–68), indicating a relatively high and positive perception of good death. The mean sub-dimension scores of the Good Death Scale were 21.73 ± 2.65 (min-max: 12–24) for “Psychosocial and spiritual”; 10.04 ± 2.04 (min-max: 3–12) for “Personal control” and 16.25 ± 2.43 (min-max: 7–20) for “Clinical control”. The total Cronbach’s Alpha coefficient of the Good Death Scale was found to be 0.84.

The mean ASAPDD score of the students is 49.21 ± 6.42 (min-max: 33–60), indicating a relatively high level of adoption of the principles of dignified death. The Cronbach’s alpha coefficient was 0.84.

The students’ mean scores for the Euthanasia Perception Scale sub-dimension were 52.89 ± 16.87 (min-max: 18–90) for “Positive attitude”, 28.44 ± 8.47 (min-max: 9–45) for “Negative attitude”, 11.64 ± 5.17 (min-max: 5–25) for “Penal attitude”, 7.05 ± 2.84 (min-max: 3–15) for “Opportunistic attitude”, and 11.50 ± 2.95 (min-max: 3–15) for “Cultural attitude”, indicating a high level of positive and cultural attitudes, a moderate level of negative attitudes, and low levels of penal and opportunistic attitudes. The Cronbach Alpha coefficient of the positive attitude sub-dimension was 0.95, the negative attitude sub-dimension was 0.91, the punitive attitude sub-dimension was 0.95, the opportunistic attitude sub-dimension was 0.81, and the “cultural attitude” sub-dimension was 0.87 (Table 2).

**Table 2:** Distribution of the scales and sub-dimensions mean scores (*n* = 259)

Scales and sub-dimensions	Number of items		Possible range	Observed range	X̄ ± SD
Good Death Scale					
Psychosocial and spiritual		9	9–36	12–24	21.73 ± 2.65
Personal control		3	3–12	3–12	10.04 ± 2.04
Clinical control		5	5–20	7–20	16.25 ± 2.43
Total		17	17–68	37–68	57.18 ± 6.97
Euthanasia Perception Scale					
Positive attitude	18		18–90	18–90	52.89 ± 16.87
Negative attitude	9		9–45	9–45	28.44 ± 8.47
Penal attitude	5		5–25	5–25	11.64 ± 5.17
Opportunistic attitude	3		3–15	3–15	7.05 ± 2.84
Cultural attitude	3		3–15	3–15	11.50 ± 2.95
ASAPDD^a^	12		12–60	33–60	49.21 ± 6.42

^a^Assessment Scale of Attitudes Toward the Principles of Dying with Dignity

### Comparison of scale scores of nursing students according to their characteristics

The mean Good Death Scale and ASAPDD scores of females were statistically significantly higher than males (*t* = 2.210, *t* = 2.323; *p* < 0.05, respectively). When considering attitudes towards euthanasia, the positive attitude sub-dimension scores of the students showed a statistically significant difference according to their age and study year (*t*=−2.066; *F* = 5.981, *p* < 0.05, respectively). The mean scores of the 24–29 age group were higher than the 19–23 age group, 2nd-year students were higher than 3rd-year students, and 4th-year students were higher than 3rd-year students. The negative attitude sub-dimension mean scores showed statistically significant differences according to their study year and knowledge about end-of-life care (*p* < 0.05). The mean scores of the 3rd-year students were higher than the 4th-year students, and those who knew end-of-life care were higher than those who did not (*F* = 4.500; *t* = 2.459, *p* < 0.05, respectively). The mean scores of the punitive attitude of the 2nd-year students were statistically significantly lower than 3rd-year students (*F* = 3.610, *p* < 0.05). The mean scores of the opportunistic attitude were statistically significantly higher for the 4th-year students than the 3rd-year students and males than females (*F* = 6.742, *p* < 0.05) (Table 3).

**Table 3:** Comparison of scale scores based on descriptive characteristics (*n* = 259)

Variables	Good Death Scale	ASAPDD	Positive attitude	Negative attitude	Penal attitude	Opportunistic attitude	Cultural attitude
	X̄ ± SD						
Age (years)							
19–23	57.16 ± 7.01	49.27 ± 6.28	52.25 ± 16.82	28.74 ± 8.55	11.73 ± 5.20	7.02 ± 2.89	11.53 ± 2.91
24–29	57.38 ± 6.62	48.61 ± 8.00	60.14 ± 16.09	25.01 ± 6.75	10.61 ± 4.89	7.42 ± 2.27	11.19 ± 3.50
Test *p*	*t*=−0.136 *p* = 0.892	*t* = 0.449 *p* = 0.653	*t*=−2.066 ***p*** **= 0.040**	*t* = 1.943 *p* = 0.053	*t* = 0.945 *p* = 0.345	*t*=−0.629 *p* = 0.530	*t* = 0.515 *p* = 0.607
**Study year**							
2nd-year^a^	56.59 ± 6.32	49.09 ± 5.87	54.82 ± 15.49	27.76 ± 8.17	10.51 ± 4.14	7.10 ± 2.69	12.01 ± 2.50
3rd-year^b^	57.88 ± 7.37	49.16 ± 6.53	47.89 ± 18.29	30.60 ± 8.97	12.60 ± 5.76	6.24 ± 2.96	11.43 ± 3.45
4th-year^c^	57.06 ± 7.17	49.39 ± 6.86	55.93 ± 15.74	26.98 ± 7.89	11.78 ± 5.31	7.79 ± 2.69	11.10 ± 2.80
Test *p*	*F* = 0.740 *p* = 0.478	*F* = 0.052 *p* = 0.949	*F* = 5.981 ***p*** **= 0.003** (a˃b; b < c)	*F* = 4.500 ***p*** **= 0.012** (b˃c)	*F* = 3.610 ***p*** **= 0**.**028** (a < b)	*F* = 6.742 ***p*** **= 0.001** (b < c)	*F* = 2.107 *p* = 0.124
**Gender**							
Female	57.78 ± 6.20	49.76 ± 5.97	52.69 ± 16.47	28.80 ± 8.28	11.72 ± 5.19	6.82 ± 2.75	11.67 ± 2.82
Male	54.94 ± 8.98	47.19 ± 7.58	53.65 ± 18.41	27.09 ± 9.08	11.33 ± 5.14	7.88 ± 3.04	10.90 ± 3.38
Test *p*	*t* = 2.210 ***p*** **= 0.030**	*t* = 2.323 ***p*** **= 0.023**	*t*=−0.373 *p* = 0.709	*t* = 1.328 *p* = 0.185	*t* = 0.491 *p* = 0.624	*t*=−2.458 ***p*** **= 0.015**	*t* = 1.702 *p* = 0.090
**Knowledge about end-of-life care**							
Yes	57.16 ± 7.04	49.77 ± 6.24	51.59 ± 16.85	29.37 ± 8.20	11.86 ± 5.16	6.82 ± 2.78	11.61 ± 2.88
No	57.21 ± 6.86	48.17 ± 6.64	55.35 ± 16.72	26.68 ± 8.72	11.21 ± 5.20	7.48 ± 2.92	11.31 ± 3.10
Test *p*	*t*=−0.053 *p* = 0.958	*t* = 1.920 *p* = 0.056	*t*=−1.714 *p* = 0.088	*t* = 2.459 ***p*** **= 0.015**	*t* = 0.964 *p* = 0.336	*t*=−1.783 *p* = 0.076	*t* = 0.787 *p* = 0.432
**Caring for a dying patient**							
Yes	56.64 ± 7.43	49.13 ± 6.47	51.03 ± 16.39	29.24 ± 8.50	12.34 ± 5.72	7.13 ± 2.92	11.30 ± 2.78
No	57.43 ± 6.74	49.26 ± 6.41	53.77 ± 17.07	28.06 ± 8.45	11.31 ± 4.88	7.01 ± 2.81	11.60 ± 3.04
Test *p*	*t*=−0.846 *p* = 0.398	*t*=−0.147 *p* = 0.883	*t*=−1.221 *p* = 0.223	*t* = 1.049 *p* = 0.295	*t* = 1.511 *p* = 0.132	*t* = 0.319 *p* = 0.750	*t*=−0.778 *p* = 0.437
**Encountering death in clinical practice**							
Yes	57.53 ± 7.19	49.21 ± 6.70	52.57 ± 17.73	28.60 ± 8.89	11.80 ± 5.44	7.14 ± 2.83	11.22 ± 2.89
No	56.83 ± 6.74	49.22 ± 6.15	53.22 ± 16.02	28.27 ± 8.05	11.47 ± 4.91	6.95 ± 2.86	11.79 ± 3.00
Test *p*	*t* = 0.808 *p* = 0.420	*t*=−0.021 *p* = 0.983	*t*=−0.309 *p* = 0.758	*t* = 0.307 *p* = 0.759	*t* = 0.512 *p* = 0.609	*t* = 0.537 *p* = 0.592	*t*=−1.569 *p* = 0.118

*ASAPDD* Assessment Scale of Attitudes Toward the Principles of Dying with Dignity, *t* Independent sample t-test, *F* One-way ANOVA

Bold values are statistically significant (*p* < 0.05)

### The correlation between the good death, ASAPDD, and euthanasia perception scale mean score

A statistically significant and positive relationship existed between the students’ ASAPDD and Good Death Scale mean scores (*r* = 0.463, *p* < 0.01) and all sub-dimensions, ranging from weak (*r* = 0.228) to moderate (*r* = 0.422) (*p* < 0.01).

There was a statistically significant, weak positive relationship between the students’ Cultural Attitude sub-dimension with ASAPDD (*r* = 0.162; *p* < 0.01), the Good Death Scale (*r* = 0.157, *p* < 0.05), the Psychosocial and Spiritual sub-dimension (*r* = 0.203; *p* < 0.01), and the Personal Control sub-dimension (*r* = 0.191, *p* < 0.01). A weak positive and statistically significant relationship existed between the students’ Positive Attitude sub-dimension and ASAPDD scores (*r* = 0.175, *p* < 0.01) (Table 4.).

**Table 4:** The relationship between students’ Good Death Scale, ASAPDD, and Euthanasia Perception Scale mean scores (*n* = 259)

		Euthanasia Perception Scale					ASAPDD
		Positive attitude	Negative attitude	Penal attitude	Opportunistic attitude	Cultural attitude	
Good Death Scale	*r*	0.006	0.029	−0.042	−0.045	0.157^*^	0.463^**^
	*p*	0.929	0.648	0.499	0.468	0.012	0.000
Psychosocial and spiritual	*r*	0.104	−0.021	−0.068	−0.012	0.203^**^	0.422^**^
	*p*	0.094	0.740	0.277	0.846	0.001	0.000
Personal control	*r*	−0.024	−0.016	−0.059	−0.063	0.191^**^	0.374^**^
	*p*	0.697	0.798	0.345	0.312	0.002	0.000
Clinical control	*r*	−0.048	0.034	−0.041	−0.063	0.083	0.228^**^
	*p*	0.439	0.584	0.509	0.311	0.180	0.000
ASAPDD	*r*	0.175^**^	−0.109	−0.111	0.082	0.162^**^	1
	*p*	0.005	0.079	0.074	0.191	0.009	

*r*= Pearson correlation coefficients, *ASAPDD* Assessment Scale of Attitudes Toward the Principles of Dying with Dignity

*Correlation is significant at the 0.05 level (2-tailed), **Correlation is significant at the 0.01 level (2-tailed)

## Discussion

This study was conducted to determine nursing students’ perceptions of a good death, their attitudes toward the principles of dignified death and euthanasia, and the relationship between them.

In this study, the mean score of nursing students on the Good Death Scale was 57.18 ± 2.65. Considering the lowest and highest scores that can be obtained from the scale, it can be said that the student’s perceptions of good death are above average and positive. Similar to our study, previous studies conducted in Türkiye found that nursing students’ perceptions of a good death were positive [1, 27]. In the studies by Yakar et al. [28] and Cevik Aktura et al. [29], the perception of good death among nursing students was high (55.66 ± 8.22 and 58.83 ± 6.12, respectively). In a mixed-methods study, Chinese nursing students had a moderate degree of good death perception [4]. The students’ positive perceptions of a good death indicate their positive perspectives on death being acceptable and dignified. A high perception of good death will provide higher clinical competence and better quality in end-of-life care [16, 17].

Nursing students’ mean score on the ASAPDD was 49.21 ± 6.42, indicating above-average and positive attitudes toward the principles of dignified death. A high level of adoption of the principles of dying with dignity among nursing students is a desirable finding that will contribute to the quality of end-of-life care. Similarly, Bilgiç [27] determined that nursing students adopted the principles of dignified death. Duru Aşiret et al. [30] found that the nursing students’ attitudes towards the principles of a good death were positive. In Üzen Cura’s study in Türkiye, nursing students’ attitudes toward the principles of dignified death were found to be moderate [31]. This study and previous studies show positive attitudes toward dignified death among nursing students. This is desirable for providing patients with higher quality and dignified end-of-life care and a good dying process [7].

Considering the lowest and highest scores that could be obtained from the subscale, nursing students’ positive and cultural attitudes toward euthanasia were above average; their negative attitudes were moderate, and penal and opportunistic attitudes were below average. The students’ highly positive attitudes indicate that euthanasia can be applied and that it is seen as a right equal to the right to life. The moderate level of negative attitudes indicates that their thoughts about euthanasia being unacceptable and constituting a violation of life are also moderate. These findings suggest that the students are not completely opposed to euthanasia, indicating their indecisiveness on the topic and a cautious approach. In the study by Karasu et al., 55.9% of health sciences faculty students believed euthanasia should not exist, 24.1% were undecided, 20% thought euthanasia should exist, and 41.1% believed euthanasia should not be legalized [19]. In another study, nursing students did not want euthanasia for themselves (60.1%) or their loved ones (65.7%) and believed that euthanasia should not be legalized in Türkiye [32]. 58.8% of nursing students did not want euthanasia to be performed for a suffering terminally ill patient, 75.3% did not want euthanasia for a suffering first-degree relative, and 60.5% did not want euthanasia to be legalized [18]. In studies conducted in Türkiye, assessments are often made using questions rather than valid and reliable scales. Although the results of these studies indicate that students generally have a negative attitude toward euthanasia, it is also seen that the proportion of students with negative attitudes is not high. These studies support our findings that students are not completely opposed to euthanasia. The fact that euthanasia is not legalized in Türkiye and that students have high cultural attitudes toward euthanasia are factors that support and explain these findings. On the other hand, nursing student’ low penal and opportunistic attitudes showed their views on the necessity of punishment and profit from euthanasia were weak. Their low punitive attitudes also show their generally positive attitudes towards euthanasia. Their low opportunistic attitudes may be a reflection of their commitment to professional values ​​and ethical responsibilities. When international studies are examined, in a study examining the attitudes of nursing students toward euthanasia following the legalization of euthanasia in Spain, the attitude of nursing students toward euthanasia was remarkably positive [9]. Examine the attitudes of a sample of Iranian Muslim nursing students towards euthanasia; 34.2% of the students had a negative attitude towards euthanasia, 41.6% had a neutral attitude, and 24% had a positive attitude [33]. Another study conducted in Iran concluded that the majority of nurses and nursing students had a positive attitude toward euthanasia [34]. The majority of Belgian nursing students expressed a high level of acceptance of euthanasia due to unbearable mental suffering and emphasized the important role of nurses in the decision-making process [35]. The majority of Dutch nursing staff expressed a desire to be involved in the decision-making processes of euthanasia [36]. Studies conducted in Belgium and the Netherlands, where euthanasia has been legalized, suggest that the attitudes of nursing students and nurses may be affected by the legal context. Differences between findings may be due to differences in political, sociocultural, and religious norms and attitude assessment tools.

The attitudes toward euthanasia are influenced by culture, laws, ethical principles, and religious beliefs [37]. In this study, the high cultural attitudes of nursing students towards euthanasia indicate that they have a high opinion of culture being effective in determining whether euthanasia is performed or not. Culture and religion have a strong relationship, and these concepts influence each other [38]. Studies conducted with university students and nursing and midwifery students have revealed that religious beliefs are an important factor and determinant in accepting euthanasia and attitudes towards euthanasia [32, 37, 39]. In a study conducted in Türkiye, most nursing students did not accept euthanasia because of conscientious discomfort and its contradiction with their beliefs [18]. In a study conducted with Muslim nursing students in Iran, more than half of the students indicated that their views on euthanasia were strongly influenced by their religious beliefs [33]. The majority of people living in Türkiye are Muslims. Islam emphasizes the sanctity of human life, and euthanasia is prohibited in Islam [33, 37]. Therefore, it can be said that culture affects attitudes towards euthanasia. Since euthanasia contradicts cultural and religious norms, positive attitudes on this issue can be difficult, and students can exhibit average and cautious attitudes on this issue.

In this study, female students had more positive perceptions of a good death and attitudes toward the principles of a dignified death, and opportunistic attitudes toward euthanasia were lower. Similarly, female Chinese nursing students received higher good death scores than males [4]. In Üzen Cura’s study [31], there was no significant difference between nursing students’ ASAPDD scores and gender. Previous studies have shown that female nursing students generally exhibit higher levels of emotional intelligence [40], empathy [41], and ethical approach [42]. These characteristics may have contributed to their greater appreciation, sensitivity, and positive attitude toward death-related issues, such as good death and the principles of dying with dignity.

In the current study, nursing students’ attitudes toward euthanasia differed according to students’ age, study level, and knowledge of end-of-life care. Students in the 24–29 age group had higher positive attitudes toward euthanasia than those in the 19–23 age group. The positive attitudes of 2nd-year students were higher than 3rd-year students, 4th-year students were higher than 3rd-year students; the negative attitudes of 3rd-year students were higher than 4th-year students; the opportunistic attitudes of 4th-year students were higher than 3rd students; and the punitive attitudes of 3rd-year students were higher than 2nd students. In a study conducted in Yangsan, nursing students with clinical experience had higher attitudes toward death and healthier perceptions of death [43]. As students approach completing their professional training, gaining professional knowledge, skills, and experience, and completing courses on professional values ​​and ethical principles, they may have enabled them to approach euthanasia from a broader perspective, from a critical and professional perspective, and to develop a balanced attitude. The negative attitudes of students who knew about end-of-life care towards euthanasia were higher than those who did not know. Students familiar with end-of-life care may have thought that euthanasia conflicted with this care process.

Students’ inability to cope with the emotional burden they experience when faced with death may cause them to distance themselves emotionally from patients, negatively affecting their end-of-life care and attitudes toward the dying patient [44]. Therefore, students need to be prepared to cope with these feelings. Studies have shown that learning about end-of-life care and palliative care is effective in reducing students’ negative emotions associated with death [45]. In our study, students experienced sadness, fear, and helplessness when they first encountered death during their clinical practice. These feelings are consistent with previous studies [44]. In a study conducted with physicians and nurses working in intensive care units and palliative care services, the participants felt sadness, helplessness, and fear when they first encountered death in their professional lives [46]. In a study examining the death experiences of Chinese nursing students in clinical practice, students experienced dread, guilt, sadness, pity, upset, and worry after the death of patients [47]. These emotions may be an indicator of students’ empathy skills and are thought to indicate the need for support mechanisms so that students can better manage the process of caring for the terminally ill. Various educational approaches can be used to address this need. Studies have shown that simulation [48, 49] and reflective writing [50] are effective in preparing students to face death and in strengthening their coping skills. In the study by Demedts et al. (2024), nursing students considered a simulation module on euthanasia to be a valuable contribution to their education [48]. Martín-Parrilla et al. found that simulation-based learning experiences were more effective than traditional classroom teaching in improving students’ ability to cope with death and in better preparing them for the challenges of end-of-life care [49]. Enabling students to participate in the care of terminally ill patients through clinical rotations and hospice/palliative care experiences may also make an important contribution to this process. In a study conducted with senior nursing students, it was found that hospice experience and student reflections led to positive changes in attitudes toward end-of-life care [51].

There was a positive relationship between the students’ attitudes toward dignified death principles and perceptions of good death. As the students’ attitudes toward principles of dignified death increase, perceptions of good death increase. Similarly, in a study conducted with nursing students, the increase in students’ perceptions of death positively affected their adoption of the principles of dignified death [27]. In a study examining the concept of dignified death in end-of-life nursing care, “good death” was determined as a positive outcome of the dignified death process [7]. In this regard, adopting the principle of dignified death may support a good death in end-of-life care.

There was a positive relationship between the students’ attitudes towards the principles of a dignified death and their cultural and positive attitudes towards euthanasia, as well as between their perceptions of a good death and their cultural attitudes towards euthanasia. As students’ acceptance of the principles of dignified death increased, their cultural and positive attitudes towards euthanasia increased. As their perceptions of good death increased, their ideas about the effectiveness of culture in euthanasia became stronger. It can be said that students’ views on death and euthanasia influence each other. This positive relationship suggests that students approach the death process with a holistic perspective in the context of ethical values ​​and cultural sensitivity. In a study conducted with intensive care nurses, it was determined that those who had a relative diagnosed with a terminal illness had more positive attitudes toward euthanasia [52]. In the study conducted with university students, a significant relationship was determined between the students’ euthanasia approaches and their attitudes towards death. As university students’ belief that death is a natural process like living increased, their positive approach to euthanasia decreased; as their tendency to see death as a release from pain increased, their positive approach to euthanasia increased. In addition, students who developed a negative attitude toward death were less likely to accept euthanasia [40]. These studies also support our study in terms of showing the relationship between the perception of death and attitudes toward euthanasia.

The findings of this study suggest that nursing students’ perceptions and attitudes toward euthanasia, good death, and death with dignity should be considered in the educational process. Because the shaping of attitudes during professional training will directly impact future clinical decisions, it is important to support students’ existing positive attitudes with education and transform them into knowledge and skills. A positive perspective on the principles of death with dignity, coupled with their lack of complete opposition to euthanasia and their ambivalence on the issue, can help students become more sensitive to patient autonomy, rights, dignity, and ethical decision-making processes. This can contribute to strengthening patient-centered care, developing ethical sensitivity, and helping them better cope with the challenges of caring for terminally ill patients. In addition to clinical experience opportunities, methods such as simulation-based training and ethical case studies can be effective in this regard.

### Limitations

The cross-sectional design and the fact that it was conducted with nursing students from only two institutions may limit the generalizability of the findings. Furthermore, the use of self-reported surveys may introduce methodological limitations, such as response bias.

## Conclusion

When the average score was evaluated, it was concluded that nursing students’ perceptions of good death and attitudes toward the principles of dignified death were above average. The students’ positive and cultural attitudes towards euthanasia were above average, their negative attitudes were at a moderate level, and their punitive and opportunistic attitudes were below average. Students experienced negative emotions in the face of death. Nursing students’ positive attitudes toward euthanasia differed by their age group; attitudes other than cultural ones differed by their study year; perceptions of a good death, attitudes toward dignified death principles, and opportunistic attitudes toward euthanasia differed by their gender, and negative attitudes toward euthanasia varied based on knowledge of end-of-life care. There was a positive relationship between students’ attitudes toward the principles of dignified death and their perceptions of a good death, as well as positive and cultural attitudes toward euthanasia. Additionally, there was a positive relationship between their perceptions of a good death and their cultural attitudes toward euthanasia.

It is recommended to comprehensively integrate end-of-life care into the nursing curriculum, use diverse teaching methods to enhance students’ understanding of death-related concepts, and provide students with care for terminally ill patients in their clinical practice. Nurse educators should consider factors that may influence students’ perceptions and attitudes toward death and euthanasia, guide students in applying the principles of a dignified and good death in their clinical practice, and also take on roles in helping students cope with the negative emotions they experience when encountering dying patients. Conducting similar studies in countries with varying cultural and religious norms will provide an opportunity to develop and compare the understanding of whether students’ perceptions and attitudes regarding death are universal or cultural.

## Supplementary Information

Supplementary Material 1.

## Data Availability

The data that supports the findings of this study are available from the corresponding author upon a reasonable request **.**.

## Abbreviations

ASAPDD: Assessment Scale of Attitudes toward the Principles of Dying with Dignity
