# Supplementary material for: Perceptions of good death and attitudes toward dignified death and euthanasia among nursing students in Türkiye: a cross-sectional descriptive-correlational study
Source: BMC Med Educ. 2025 Dec 17;26:115. doi: 10.1186/s12909-025-08435-6 (PMC12822026; doi:10.1186/s12909-025-08435-6)
Supplement: Supplementary file 1 — Supplementary Material 1. [file 12909_2025_8435_MOESM1_ESM.pdf]

Personal Information Form

Number: **Date:**

1. Age:
2. Study year:

   ( ) 2nd year ( ) 3rd year ( ) 4th year

3. Gender:

   ( ) Female ( ) Male

4. Do you have knowledge about end-of-life care?

   ( ) Yes ( ) No

5. Have you provided care to a dying patient during your clinical practice?

   ( ) Yes ( ) No

6. Have you encountered death during your clinical practice?

   ( ) Yes ( ) No

7. If your answer is “Yes”:

   How did you feel when you first encountered death? (You may select more than one.)

   ( ) Despair

   ( ) Fear

   ( ) Sadness

   ( ) Other: …………………
